# Cohort profile: the Nigerian HIV geriatric cohort study

**DOI:** 10.1186/s12889-020-09833-9

**Published:** 2020-11-26

**Authors:** Patrick Dakum, Yohanna Kambai Avong, Michael Kolawole Odutola, James Okuma, Gbenga Ayodele Kayode, Iboro Ekpo Nta, Nicaise Ndembi, Charles Mensah, Hadiza Khamofu, Prosper Okonkwo, John Oko Okpanachi, Echezona Ezeanolue

**Affiliations:** 1 Institute of Human Virology, Abuja, Nigeria; 2 FHI360, Abuja, Nigeria; 3 APIN Public Health Initiatives, Ltd/Gte, Abuja, Nigeria; 4 Catholic Caritas Foundation of Nigeria, Abuja, Nigeria; 5 Nigeria Implementation Science Alliance, Abuja, Nigeria; 6 Department of Paediatrics and Child health, University of Nigeria, Enugu, Nigeria

**Keywords:** Elderly, Geriatric, HIV/AIDS, Nigeria

## Abstract

**Background:**

The Nigerian HIV Geriatric Cohort (NHGC) is a longitudinal cohort setup to learn how elderly people living with HIV (EPLHIV) in Nigeria fare, despite not being prioritized by the national treatment program, and to deepen knowledge for their differentiated care and achieve better outcomes. In this paper, we describe data collected on sociodemographic and clinical data from EPLHIV from the inception of Nigeria’s national HIV program to 2018.

**Methods:**

Patient-level data spanning the period 2004 to 2018, obtained from comprehensive HIV treatment hospitals, that are supported by four major PEPFAR-implementing partners in Nigeria were used. These 4 entities collaborated as member organizations of the Nigeria Implementation Science Alliance. We defined elderly as those aged 50 years and above. From deidentified treatment records, demographic and clinical data of EPLHIV ≥50-year-old at ART initiation during the review period was extracted, merged into a single REDcap® database, and described using STATA 13.

**Results:**

A total of 101,652 EPLHIV were analysed. Women accounted for 53,608 (53%), 51,037 (71%) of EPLHIV identified as married and 33,446 (51%) unemployed. Median age was 57.1 years (IQR 52–60 years) with a median duration on ART treatment of 4.1 years (IQR 1.7–7.1 years). ART profile showed that 97,586 (96%) were on 1st-line and 66,125 (65%) were on TDF-based regimens. Median body mass index (BMI) was 22.2 kg/m^2^ (IQR 19.5–25.4 kg/m^2^) with 43,012 (55%), 15,081 (19%) and 6803 (9%) showing normal (BMI 18.5 – < 25 kg/m^2^), overweight (BMI 25 - < 30 kg/m^2^) and obese (BMI ≥30 kg/m^2^) ranges respectively. Prevalence of hypertension (systolic-BP > 140 mmHg or diastolic-BP > 90 mmHg) was 16,201 (21%). EPLHIV median CD4 count was 381 cells/μL (IQR 212–577 cells/μL) and 26,687 (82%) had a viral load result showing < 1000copies/ml within one year of their last visit. As for outcomes at their last visit, 62,821 (62%) were on active-in-treatment, 28,463 (28%) were lost-to-follow-up, 6912 (7%) died and 2456 (3%) had stopped or transferred out. Poor population death records and aversion to autopsies makes it almost impossible to estimate AIDS-related deaths.

**Conclusions:**

This cohort describes the clinical and non-clinical profile of EPLHIV in Nigeria. We are following up the cohort to design and implement intervention programs, develop prognostic models to achieve better care outcomes for EPLHIV. This cohort would provide vital information for stakeholders in HIV prevention, care and treatment to understand the characteristics of EPLHIV.

## Why was the cohort set up?

Older adults living with the human immunodeficiency virus (HIV) are set to multiply worldwide [1–3]. HIV/AIDS is a major public health problem among older adults, approximately 6 million people over the age of 50 years are living with HIV worldwide [4]. In sub-Saharan Africa, approximately 3 million people older than 50 years of age, are HIV-infected, which represents an average of 14% of affected adults and 11% of adults on antiretroviral therapy (ART) [5, 6]. In Nigeria, a cross-sectional study at one of the largest treatment centres, reported that 10% of the studied population were aged, 51 to 60 years, while 0.6% were > 60 years [7]. At the national level, 54% of the 3.4 million people that were living with HIV in 2012 were between 15 to 64 years [8]. The recently concluded 2018 Nigeria HIV/AIDS Indicator and Impact Survey (NAIIS), a household-based survey that assessed the prevalence of HIV and related health indicators in household members aged 0–64 years old, found the distribution of HIV burden across those aged 50 years and above at 13%, which is approximately 280,000 based on new total estimates of 1.9 million persons living with HIV in Nigeria [9]. Based on the NAIIS, gender disaggregation statistics showed that prevalence was highest among females aged 35–39 years at 3.3%, and the highest among males age 50–54 years at 2.3%. This pool will grow significantly as many ART patients are expected to cross the 50-years baseline, given the efficacy of ART and the current global drive to expand access to treatment and end HIV/AIDS by 2030 [10].

Information on the geriatric population living with HIV in Nigeria is sparse and a systematic description of the HIV geriatric cohort is required to change this. The early and widely recognized impact of AIDS on the elderly was the phenomenon of AIDS orphans— children who lost at least a parent as a result of HIV-related mortality. The report *Children on the Brink: 2002* indicates that in 2001, there were 38 million orphans in Africa, 11 million of whom were attributable to AIDS mortality [11]. It predicted that in 2010, 42 million orphans will be in Africa, 20 million of whom will be the result of AIDS mortality. Since formal social security systems do not exist in most sub-Saharan African countries, the elderly largely bear the burden of taking care of the orphans, which compromises their health [12].

HIV-infection and attendant antiretroviral (ARV) treatment complicate elderly health. AIDS has a disproportionate impact on the health of the elderly compared with the younger adults [13]. Opportunistic infections (OIs) such as pneumocystis pneumonia, extra-pulmonary tuberculosis, and candidiasis have been reported in the elderly [13]. Both HIV and aging synergistically decrease function of B and T-cell lymphocytes which compromises the host’s immunity leading to more OIs. Also, HIV-infection independently creates metabolic abnormalities [14, 15] and the toxicities of ARVs and certain drugs used for OIs, create a wide range of diseases including, type 2 diabetes mellitus, myocardial infarction (MI) and atherosclerotic cardiovascular disease [16, 17]. In 2016, a Médecins Sans Frontières (MSF) multi-national study reported a higher risk of death among HIV infected people who were aged 50 years and above [16]. Furthermore, age is a risk factor for cardiometabolic disorders thus, older adults are likely to bear a dual burden of HIV and cardio-metabolic abnormalities [18], which is on the rise in sub-Saharan Africa.

Nigeria’s HIV-infected elderly face significant challenges due to age, inadequate health care, low social security and a dwindling economy. Although this context necessitates a concerted focus on the elderly, there is no structured care for them. Several reasons contribute to this; earlier studies projected HIV/AIDS prevalence and transmission as a youth issue [2], this has inadvertently omitted elderly-care in the HIV-control program design. Being elderly is associated with sustaining risky behaviour like less condom use, higher preference for multiple sexual partners [19] and wife inheritance [20], which predisposes to new infections [21, 22].

It is therefore critical to establish a HIV geriatric cohort in Nigeria to provide evidence to add life to years [23], improve quality of life and outcomes of older adults living with HIV. In this paper, we describe socio demographic and clinical data from older HIV positive clients from the inception of Nigeria’s national HIV program, and we plan to longitudinally study this cohort with the aim of designing and implementing intervention programs that would improve better health outcomes.

### Which cohorts contribute to the collaboration?

The treatment and prevention of HIV/AIDS in Nigeria is coordinated by the National Agency for the Control of AIDS (NACA) – an agency of the Federal Ministry of Health (FMOH). The United States of America’s Government (USG) since 2006, through its Presidents Emergency Plan for AIDS Relief (PEPFAR), supported Nigeria’s free access ART program. Four major implementing partners (IPs) directly cover the treatment program across all of Nigeria’s six geopolitical regions in partnership with health facilities under the general supervision of the US agencies and NACA.

These IPs provide technical support for HIV treatment in most Nigerian states, they include: the Institute of Human Virology Nigeria (IHVN) covering the Federal Capital Territory (FCT) and 3 States (Kano, Katsina and Nassarawa); FHI360 covering 12 States (Adamawa, Akwa Ibom, Anambra, Bauchi, Bayelsa, Borno, Cross River, Edo, Jigawa, Lagos, Rivers and Yobe); APIN Public Health Initiative in Nigeria (APIN) covering 8 States (Lagos, Ogun, Osun, Ekiti, Ondo, Oyo, Plateau and Benue); and Catholic Caritas Foundation of Nigeria (CCFNG) covering 4 States (Delta, Ebonyi, Enugu and Imo). These IPs use a nationally standardized method of data collection and schedule monthly follow-up visits for their patients. This cohort is the first to be set-up to pool geriatric data from these IPs. Furthermore, to the best of our knowledge, no study has described Nigeria’s nationwide geriatric HIV positive population using combined data from these IPs.

### Definition of terms

#### Older age

The statistical cut-off for assessing old age varies. Rather than artificially categorizing life into stages such as middle age or old age, it assumes we age from birth. Nevertheless, for statistical purposes, it is often necessary to divide populations into age groups. World Health Organization (WHO) recommends 60 years and over as a statistical cut-off for older age [23]. However, some analyses refer to populations of different ages such as ≥50 years, ≥ 65 years or ≥ 80 years. In this study, we adopted ≥50 years as our cut-off for old age. This assumption is based on Nigeria’s population life expectancy from birth (2016) which was 54 years [men (55 years); women (56 years)] [24]. Participants who are ≥50 years at time of testing positive to HIV will be classified as ‘older adults’, ‘elderly’ or ‘geriatrics’.

#### WHO stage 3 and 4

The diseases defined as WHO stage 3 and 4 in this study are presented in Table 2 (pages 28 to 29) of the National Guidelines for HIV Prevention Treatment and Care (2016, 8].

#### Lost to follow-up (LTFU)

LTFU was defined as > 90 days late for the next scheduled appointment, with visits scheduled every month. Participants could experience multiple LTFU episodes after returning to care in the interim. We report on LTFU event at the last visit based participants status at their last vist.

### Who is in the cohort?

The study population included all EPLHIV from four major IPs providing treatment services across all of Nigeria’s six geopolitical regions. Patient-level socio demographic and clinical data of participants treated at the health facilities supported by the IPs were used. Patients were prospectively enrolled at first contact with a HIV-service facility and are followed-up monthly. We use de-identified data of EPLHIV from the date first tested positive for HIV as contained in the IPs databases between the period 2004–2018. The databases were merged as a single REDcap® database and analysed using STATA 13. Ethical clearance for the use of the secondary data was obtained from the IRB board (IRB019-SD) of APIN.

### How often is follow up?

Data are collected during the initial visit (study enrolment) and atleast monthly during follow up. Research health care workers collect epidemiological data using a standardized uniform data collections tools. Detailed contact information (address and phone numbers) are collected from all participants. Follow-up visits are scheduled at appropriate times and reminders are sent by text messages and phone calls. Where participants cannot be reached by phone, home visits are conducted.

### What has been measured?

The data are collected at EPLHIV’s clinic visit using national standardized Case Report Forms (CRFs). Data was collected at baseline and scheduled follow-up visits on socio demographic (sex, age, marital status, education, occupation), dates of HIV diagnosis and initiating therapy, AIDS-defining conditions, laboratory data (CD4 cell count, viral load), ART regimen and others to determine HIV comorbidities (blood pressure, body mass index, blood glucose measurements). Trained clinicians obtained this information at each patient’s visit. The HIV biomarkers including CD4 cell count done 6-monthly and since 2016 routine viral load done 6 months post-initiation of ARTs for newly identified clients, then yearly for stable patients (those with VL < 1000copies/ml) and more frequently as needed for patients with derailed values. The CRFs are entered into a secure electronic capture database with a study ID used as a unique patient identifier. Data was cleaned and stored in a central geriatric database. In the analysis, we report baseline characteristics, treatment and clinical outcomes as percentages for categorical data and medians (interquantile ranges) for continuous data.

Participants were considered on ART at baseline if they initiated before or within 30 days of enrolment. CD4 count, BMI, weight, WHO stage and blood pressure (BP) were those measurements closest to enrolment, provided within 30 days.

Outcomes reported are duration on ART in years from the time of enrolment to the last ART visit. We also report on EPLHIV’s ART regimen, participant status (active in care, LTFU, deaths, transferred out/stopped) and HIV viral load (measured within one year) at time of last ART visit.

### What has been found?

**Fig. 1:**
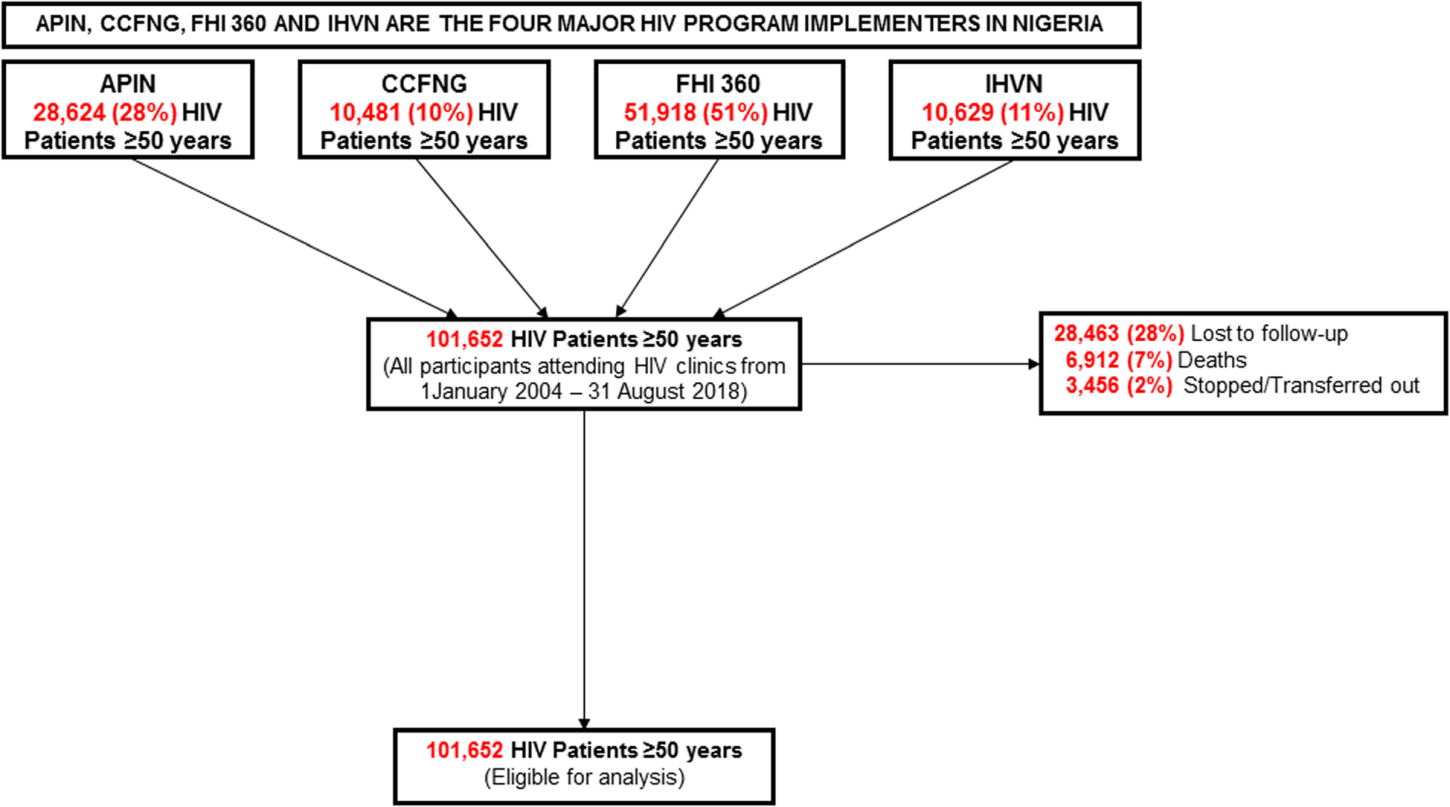
Study participants flowchart

Figure 1 shows that a total of 101,652 NHGC participants [APIN = 28,624 (28%), CCFNG = 10,481 (10%), FHI360 = 51,918 (51%) and IHVN = 10,629 (11%)] were enrolled for treatment at the health facilities supported by the IPs in 28 of the 36 States in Nigeria and the FCT, Abuja between 2004 and 2018. The NHGC includes approximately 36% of estimated EPLHIV in Nigeria.

The baseline characteristics of NHGC participants socio demographic and clinical findings are presented in Table 1. Median age was 57.1 (IQR 52–60 years); majority were women 53,608 (53%) and married 51,037 (71%). Participants data showed atleast secondary school education attendance by 31,280 (52%) and unemployment in about 33,446 (51%); most of the participants (73%) commenced ART at WHO stage I/II; Median body mass index (BMI) at enrolment was 22.2 kg/m^2^ (IQR 19.5–25.4 kg/m^2^). 43,012 (55%) had normal BMI (18.5 kg/m^2^–24.9 kg/m^2^), but 15,081 (19%) were overweight with a BMI of 25- < 30 kg/m^2^ and a few were obese 6803 (9%) with a BMI of ≥30 kg/m^2^. Median systolic and diastolic blood measurements were 120 mmHg (IQR 110–137 mmHg) and 80 mmHg (IQR 70–89 mmHg) respectively; 16,201 (21%) of patients had hypertensive readings (systolic blood pressure > 140 mmHg or diastolic blood pressure > 90 mmHg). Median CD4 count was 381 cells/μL (IQR 212–577 cells/μL).

**Table 1:** Baseline characteristics of NHGC participants

Characteristics	All participants *N* = 101,652
Socio-demographics	
Age (years), median (IQR), *n* = 101,652	57.1 (52–60)
Age categories (years), n(%)	
50–59	72,894 (72%)
60–69	22,740 (22%)
70 and above	6018 (6%)
Missing	0 (0%)
Gender, n(%)	
Female	53,608 (53%)
Males	48,044 (47%)
Missing	0 (0%)
Marital status, n(%)	
Single	3493 (6%)
Married	51,037 (71%)
Widowed/Separated/Divorced	16,944 (24%)
Missing	30,178 (30%)
Education, n(%)	
None	11,657 (19%)
Primary	17,842 (29%)
Secondary	19,768 (33%)
Post – secondary	11,512 (19%)
Missing	40,873 (40%)
Occupation, n(%)	
Unemployed	33,446 (51%)
Employed	29,977 (46%)
Retired	18,32 (2.8%)
Missing	36,397 (36%)
Service Entry Point, n(%)	
VCT	53,421 (63%)
HCT	14,920 (63%)
Outpatient	5227 (22%)
Transfer In	1671 (7%)
In-patient	692 (3%)
Others	1337 (6%)
Missing	24,384 (24%)
AM	
APIN	28,624 (28%)
CCFNG	10,481 (10%)
FHI360	51,918 (51%)
IHVN	10,629 (11%)
Missing	0 (0%)
Clinical	
WHO stage, n(%)	
I	26,617 (47%)
II	14,500 (26%)
III	13,728 (24%)
IV	2102 (4%)
Missing	44,705 (44%)
Weight, Kg, median (IQR), *n* = 79,912	59 (50–68)
Systolic Blood Pressure (SBP), mmHg, median (IQR), *n* = 76,421	120 (110–137)
Diastolic Blood Pressure (DBP),mmHg,median (IQR), *n* = 76,421	80 (70–89)
Hypertension (SBP > 140/DBP > 90 mmHg, n(%), *n* = 76,421	16,201 (21%)
Body Mass Index, Kg/m^2^, median (IQR), *n* = 78,104	22.2 (19.5–25.4)
Body Mass Index, Kg/m^2^, n(%)	
< 17	6240 (8%)
17- < 18.5	6967 (9%)
18.5 – < 25	43,012 (55%)
25 – < 30	15,081 (19%)
30 and above	6803 (9%)
Missing	23,548 (23%)
CD4 cell count (cells/μl), median (IQR), *n* = 66,027	381 (212–577)
CD4 cell count < 200, cells/μl, n(%) ,*n* = 66,027	15,325 (23%)
CD4 cell count < 500, cells/μl, n(%), *n* = 66,027	43,783 (66%)

Results are number and column % of those with non-missing data; missing data rows are number and column % of missing data

*IQR* Interquartile range, *ART* Antiretroviral Therapy, *APIN* APIN Public Health Initiative in Nigeria, *CCFNG* Catholic Caritas Foundation of Nigeria, *IHVN* Institute of Human Virology Nigeria

Results are number (column percentage of those with non-missing data) for categorical variables and median (interquartile range) for continuous variables

Table 2 present treatment outcomes of NHGC participants at their last visit. The median duration on ART treatment was 4.1 years (IQR 1.7–7.1 years); majority were on 1st line regimen at their last ART visit 97,586 (96%) – the TDF based regimen had the highest number of patients 66,125 (65%), consistent with the requirement of the treatment guidelines. Cohort’s most patients (82.3%) had viral suppression less than 1000 copies/ml within one year of their last visit. In terms of treatment outcome at their last visit, 62,821 (62%) were active in treatment; 28,463 (28%) lost to follow-up; 6912 (7%) died, while on treatment and 3456 (3%) had stopped or were transferred out.

**Table 2:** Outcomes of NHGC participants at last clinic visit

Outcome	All participants N = 101,652
ART Regimen	
ART regimen line at last visit, n(%)	
First line	97,586 (96%)
Second Line	4066 (4%)
Current ART regimen at last visit, n(%)	
ABC-Based	3050 (3%)
AZT-Based	29,479 (29%)
TDF-Based	66,125 (65%)
Others	2998 (3%)
Number of years on ART treatment, median (IQR)	4.1 (1.7–7.1)
Number of years on ART treatment, n(%)	
< 2	29,225 (29%)
2 - < 5	31,095 (31%)
5 - < 10	31,512 (32%)
10 and above	9819 (10%)
Viral Load^a^	
Viral load at last measurement, copies/ml, n(%)	
< 1000	26,687 (82%)
Participant status	
Status at last visit, n(%)	
Active in care	62,821 (62%)
Died	6912 (7%)
Lost to follow-up	28,463 (28%)
Stopped/Transferred Out	3456 (3%)

Results are number and percent of those with non-missing data; ^a^Only 38,199 (38%) had a viral measurement within one year of their last visit

*ART* Antiretroviral Therapy, *ABC* Abacavir, *AZT* Zidovudine, *TDF* Tenofovir Disoproxil Fumarate

## Discussions

This study described the characteristics of EPLHIV in Nigeria; we found a lower prevalence of hypertension in older people on ART in Nigeria as compared to the prevalence of hypertension in sub-Saharan Africa (31.9, 95%CI 18.5–49.2%) and the global prevalence of hypertension (42.0, 95%CI 29.0–55.4%) [25]. Despite the relatively low prevalence of hypertension in older people living with HIV in Nigeria, it is very important for public health experts to deploy preventive interventions that will reduce the occurrence of hypertension. Also, government, HIV program implementers, donors and other HIV stakeholders should ensure that older people on ART who are living with high blood pressure should be able to receive care for hypertension when they visit HIV clinic. In addition, we observed that four out of five older people commenced on ART, were achieving viral suppression which is commendable. This estimate is very similar to the national prevalence of viral suppression among the total number of people placed on ART in Nigeria [26]. Our finding is consistent with the regional prevalence of viral suppression among people on ART in West and Central Africa region [27] but lower than the observed prevalence in East and Southern Africa region [28]. However, despite the high prevalence of viral suppression among patients on ART in Nigeria, including most African countries, more effort should be geared towards improving HIV identification as less than two-thirds of this populations are aware of their HIV status [26].

### Future plans?

The NHGC is the largest cohort of EPLHIV in Nigeria; it provides a strong base for future studies on comorbidities associated with aging in EPLHIV in the country. We plan to follow up this cohort and obtain data that would be used to implement intervention programs, develop prognostic models and predict better outcomes for EPLHIV using multi-variable indices.

### What are the main strengths and weaknesses?

A large pool of geriatric patients and the collaborating spirit of the IPs contributing data to this cohort is a key strength for the NHGC. The substantial dataset of the NGHC permits examination of prognoses in improving the quality of life of EPLHIV. Data of patients in this cohort are collected during routine clinical visits, and incomplete data from missing variables exist. Data on AIDS- mortality is likely under-reported as ART clients that died outside hospital settings may never have this status reverted to their service provider except following active tracking. Furthermore, public mortality repositories in Nigeria do not usually share information with hospitals and clinical autopsies are not mainstream [29, 30].

Another expected limitation of a longitudinal cohort study is loss to follow-up, a particularly challenging norm in low income settings where participants have a poor history of follow-up in clinical care. To improve patient’s retention in our cohort, we developed a monitoring and tracking system to identify patients that are due for follow up visits. These patients are called using registered anonymous public health lines and sent cyphered short message service prompts to remind them of their clinic visits. We also deployed community engagement strategies and care group network to keep the patients engaged in care.

## Conclusion

The NHGC represents about 36% of the total estimates and is the largest cohort of EPLHIV in Nigeria. It provides a strong basis for studies on comorbidities associated with aging in EPLHIV in the country. Longitudinal monitoring of this cohort could be used to design and implement intervention programs, develop prognostic models and achieve better care outcomes for EPLHIV.

## Data Availability

The chief investigator of the study is Patrick Dakum, whose e-mail contact is (pdakum@ihvnigeria.org). The data remains the property of the contributing NISA member organizations, whose representatives manage the NHGC via a steering committee of the collaboration. Inquiries on the use of collected data are welcomed and encouraged and all proposals for specific analyses are to be reviewed by a scientific committee. The datasets are available from the first author on reasonable request.

## Abbreviations

HIV: Human immunodeficiency virus
ART: Antiretroviral therapy
AIDS: Acquired immune deficiency syndrome
NAIIS: Nigeria HIV/AIDS indicator and impact survey
ARV: Antiretroviral
OIs: Opportunistic infections
IM: Myocardial infarction
MSF: Médecins Sans Frontières
NACA: National Agency for the Control of AIDS
FMOH: Federal Ministry of Health
USG: United States of America’s Government
PEPFAR: its Presidents Emergency Plan for AIDS Relief
IPs: Implementing partners
IHVN: Institute of Human Virology Nigeria
FCT: Federal Capital Territory
APIN: Public Health Initiative in Nigeria
CCFNG: Catholic Caritas Foundation of Nigeria
CDC: Centers for Disease Control and Prevention
USAID: United States Agency for International Development
DoD: Department of Defense
PLHIV: People living with HIV
CRFs: Case Report Forms
VL: Viral load
DTG: Dolutegravir
BMI: Body mass index
IQR: Interquantile range
EPLHIV: Elderly people living with HIV
NHGC: Nigerian HIV Geriatric Cohort.
